# TAK1 inhibition increases proliferation and differentiation of chick retinal cells

**DOI:** 10.3389/fcell.2022.698233

**Published:** 2022-09-13

**Authors:** Casandra Carrillo, Vagisha Ravi, Sarika Tiwari, Ellen A. Chernoff, Teri L. Belecky-Adams

**Affiliations:** Department of Biology, Indiana University-Purdue University Indianapolis, Indianapolis, IN, United States

**Keywords:** TGF-β-activated kinase 1, TAK1, MAP3K7, retina, chick, proliferation, differentiation

## Abstract

The factors necessary for the differentiation of cell types within the retina are incompletely understood. The transforming growth factor beta (TGF-β) superfamily, including TGF-β1 and 2, the bone morphogenetic proteins, and the activins have all been implicated in differentiation; however, the mechanisms by which these factors affect differentiation are only partially understood. The studies herein focus on a potential role for transforming growth factor β-activated kinase 1 (TAK1), a hub kinase that lies at the intersection of multiple signaling pathways, in the differentiation of cell types within the chick retina. Previous studies have focused predominantly on the role this kinase plays in the inflammation process and axonal growth. TAK1 is downstream of multiple signaling pathways that are critical to development of the central nervous system, including transforming growth factor β (TGFβ), bone morphogenetic proteins (BMPs), and activins. The present study indicates that activated TAK1 is found throughout the developing retina; however, it is localized at higher levels in dividing and differentiating cells. Further, *ex ovo* retinal studies using TAK1 inhibitor 5Z-7-oxozeaenol increased both progenitor and differentiating cell populations, accompanied by a substantial increase in proliferation and a smaller increase in cell death. These results indicate a unique role for TAK1 in differentiating and proliferating retinal cells.

## Introduction

The hierarchical organization of the mature neural retina, containing seven basic cell types, is common to vertebrate retinas (Turner and Cepko, 1987; Holt et al., 1988). The developing retina is comprised of homogeneous group of cells, retinal progenitors, which generate groups of cell types at specific stages throughout development (Wetts and Fraser, 1988; Turner et al., 1990). In most animals, cell cycle length is known to increase throughout development, increasing the length of G_1_ and increasing the overall time of cell division (Arai et al., 2011; Ferree et al., 2016). Many studies have confirmed that retinal progenitors are mostly multipotent. However, progenitors change in their capacity to generate earlier-born cells at later stages of development (Turner and Cepko, 1987; Jensen and Raff, 1997; Cepko 1999). Differentiation of retinal cell types is the result of signaling pathways and transcription factors activity which regulate cell type specification (Ohnuma et al., 1992; Tomita et al., 1996; Cepko 1999; Hatakeyama and Kageyama 2004). Likewise, the activation of specific signaling pathways and cell cycle genes is essential for cells that remain in the cell cycle. Many of the key questions that remain lie in the regulation and coordination of proliferation, cell cycle exit, and differentiation, and the signaling pathways that govern the processes.

When cell cycle exit and differentiation do not occur properly, issues such as hyperplasia and lack of differentiated cell types lead to improper function of the retina (Miles and Tropepe 2016). Across various species, multiple factors have been identified that play a role in cell cycle exit, including extrinsic factors, signaling pathway members, and transcription factors (Dyer and Cepko, 2001; Miles and Tropepe 2016). In addition to this, there are factors that are associated with shortening of the cell cycle such as transcription factor *E2f1,* cyclin G2 (Arai et al., 2011) and factors associated with the lengthening of the cell cycle such as *Erf* and *Fbxw7* (Arai et al., 2011). Transforming growth factors β (TGFβs), activins, and bone morphogenetic proteins (BMPs) are all secreted factors that have been implicated in cell cycle exit and differentiation (Chen et al., 2002; Roy and Ingham 2002; Vogel et al., 2010; Jarrin et al., 2012; Wagner et al., 2017).

When it comes to cell cycle exit and differentiation, there is evidence to suggest that it is more complex than previously shown in retinal development, which indicates that there may be influential signaling factors yet to be discovered (Dyer and Cepko, 2001). Although the TGF-β superfamily and the downstream canonical signaling pathways that support them have been investigated, there has been little evaluation of non-canonical pathways. The canonical signaling pathways primarily involve the activation of SMAD family members which, when activated, form heteromers that are transported to the nucleus to act as transcription factors. The most actively studied non-canonical pathway of the TGF-β superfamily involves the activation of a mitogen-activated protein kinase (MAPK) pathway. Receptors for each pathway activates TAK1 (also known as MAP3K7), followed by the activation of downstream kinases and transcription factors (Kim and Choi 2012; Shi and Sun 2018).

TAK1 is a serine-threonine kinase that acts as a hub kinase combining extracellular signaling events from many receptors and concentrates those signals on the regulation of mitogen activated kinase 3/6 (MKK3/6), MKK4/7, and IκB kinase (IKK) (Ninomiya-Tsuji et al., 1999; Munoz-Sanjuan et al., 2002; Blonska et al., 2005; Sato et al., 2005). It has been determined that MKK3/6 regulates p38, MKK4/7 activates c-jun N-terminal kinase (JNK), and phosphorylation of IKK leads to the destruction of IκB, allowing NFκB to be transported to the nucleus (Choi et al., 2012). JNK and its upstream regulators MKK4/7 are key regulators of retinal ganglion cell death in vertebrates (Hong et al., 2007; Choi et al., 2012). A recent study in retinal development showed that a loss of both MKK4 and MKK7 led to a decrease in JNK signaling and a subsequent decrease in retinal ganglion cells (Syc-Mazurek et al., 2018). While the role of JNK, MKK4, and MKK7 have been investigated, the role of TAK1 in retinal development is yet to be determined.

The study described herein investigates the role of TAK1 in chick retinal development. Using immunofluorescence, we describe localization patterns of activated phospho-TAK1 (pTAK1) at critical stages of retinal development from the optic cup up to embryonic day 18 (E18). To investigate a potential role for TAK1 in retinal development, embryonic day 6 (E6) *ex ovo* retinal cultures were treated with vehicle or TAK1 inhibitor (5Z)-7-Oxozeaenol. Quantitation of retinal cell types 24 h following treatment indicated an increase in both differentiated *and* proliferating cells with a small increase in cell death. The results of this study have led us to hypothesize a dual role for TAK1 in retinal development; TAK1 does appear to play a role in differentiation; however, we have identified a novel role for TAK1 in the proliferation of retinal progenitor cells.

## Materials and methods

### Tissue collection and processing

Fertilized white leghorn chicken eggs were procured from University of Michigan and stored at 16°C in a BOD (Biological Oxygen Demand) incubator (Jeio tech: IL-11A). Eggs were incubated at 37°C for the specified periods of time (Kuhl, Flemington, NJ). Eyes used for immunohistochemistry were enucleated, extraocular tissues removed in 1X phosphate-buffered saline (PBS) and fixed overnight in 4% paraformaldehyde diluted in 0.1M phosphate buffer, pH7.4. Samples were rinsed twice in 1X PBS at room temperature incubated overnight with increasing concentrations of sucrose solutions in order: 5%, 10%, 15%, and 20% sucrose made in 0.1M phosphate buffer, pH 7.4. Samples were then placed in 20% sucrose and optimal cutting temperature (OCT) medium at a ratio of 3:1 (4583 Tissue-Tek, Sakura, Torrance CA). The samples were oriented in the 3:1 solution oriented so that sections would be taken through the naso-temporal axis. They were then placed in dry ice to freeze and stored for future sectioning at −80°C. Samples were sectioned using a Leica CM3050 S cryostat at a thickness of 12 µm and placed on vectabond-coated (Vector Labs, Burlingame, CA) superfrost plus slides (Fisher Scientific, Pittsburgh, PA) and then stored at −80°C.

### Immunohistochemistry

Slides with cryosections previously stored at −80°C were thawed to room temperature (RT) and the perimeter of the sections was lined using a peroxidase anti-peroxidase (Tsalamandris et al., 2019) pen (Electron Microscope Sciences, Hatfield, PA, Cat #71310). All immunohistochemistry steps were conducted at RT in a humid chamber lined with wet filter papers covered by nylon mesh unless otherwise noted. Tissue samples were fixed with 4% paraformaldehyde made in 0.1M phosphate at pH7.4 for 30 min and then rinsed twice with 1X PBS for 2 minutes. Sections were permeabilized with methanol for 10 min and subsequently rinsed with 1X PBS. Antigen retrieval was conducted by treating sections with 1% SDS in 0.01M phosphate buffer (Fisher Scientific, Pittsburgh, PA) for 5 min and then rinsed for 5 min three times with 1X PBS. Tissue was rinsed twice with 1X borate buffer at pH 8.0 for 5 min and then rinsed twice with 1X PBS for 5 min. Following these steps, sections were then treated with fresh 1% sodium borohydride (Acros, New Jersey) in 1X PBS for 2 min to reduce auto fluorescence. Sections were then blocked with 10% donkey or goat serum for 30 min. After blocking, primary antibodies were diluted into a cocktail of 0.025% triton-X diluted into 1X PBS and 2% of goat or donkey serum to their respective concentrations and added to sections. Sections were incubated with primary antibody solution overnight at 4°C. Primary antibodies that were amplified with biotin-streptavidin were used at half of their normal concentration and incubated in the same fashion. After overnight incubation with primary, samples were rinsed using 1X PBS twice before being treated with secondary antibody or a biotinylated antibody. Secondary antibodies were diluted with 1X PBS at their respected concentrations and placed on slides incubated at room temperature for 1 h in the dark. Subsequently, sections were rinsed with 1X PBS twice for 2 min before being incubated with 2 μg/ml Hoechst stain (Invitrogen, Cat #H1399) for 2 minutes. For samples subjected to biotin amplification, samples were treated with DyLight conjugated streptavidin (DyLight 549, Vector Labs, Burlingame, CA, Cat# SA-5549-1) for an additional hour followed by a rinse with 1X PBS and incubation for 2 min with Hoechst. Finally, sections were rinsed twice more with 1X PBS and then coverslipped with aqua polymount (Polyscience Inc., Warrington, PA, Ca#18606-20). The edges of the coverslips were sealed with clear nail polish (Electron Microscopy Sciences, Hatfield, PA, Cat #71310).

For co-labels with antibodies against phospho TAK1 (pTAK1) and other antibodies raised in rabbit, citrate antigen retrieval with tyramide amplification was done following permeabilization with methanol. Briefly, sections were incubated with 10 mM sodium citrate with 0.05% Tween pH 6.0for 1 h at 63°C, then allowed to cool for 20 min followed by two rinses of with 1X PBS. Sections were subsequently treated for 2 min with 1% sodium borohydride (Acros, New Jersey) in 1X PBS and then blocked with 10% donkey or goat serum in 1X PBS containing 0.25% Triton X-100 for 30 min. Primary antibodies were then diluted 5 to 10 times normal concentration in a solution of 2% donkey or goat serum and 0.025% triton X-100 diluted in 1X PBS. After incubation overnight at 4°C, sections were rinsed with 1X PBS and incubated with 3% solution of hydrogen peroxide in methanol for 15 min. Sections were washed with 1X PBS twice for 2 min before being incubated with biotinylated secondary antibody (Vector Labs, Burlingame, CA) in 1X PBS in the dark for 1 hour at room temperature. Sections were then washed using HRP streptavidin (Vector Labs, Burlingame, CA) in 1X PBS 1:1000 (and 1:500 for pSMAD1) for an hour in the dark. Afterwards, slides were then washed twice in 0.1M Tris-HCl, 0.15M NaCl, 0.05% Tween-20 (TNT) buffer and incubated with fluorescein plus amplification reagent diluted 1:300 in 1X tyramide signal amplification (TSA) amplification diluent (Perkin Elmer, Waltham, MA, Cat# NEL741001KT) for 5 min in the dark. Slides were subsequently washed twice in TNT buffer followed by two 2 min rinses in 1X PBS, counterstained with Hoechst solution and mounted with aqua polymount and cover slipped. All images were acquired using an Olympus Fluoview FV 1000 confocal microscope.

### Western blots

Chick retinas were dissected out and placed in lysis buffer (5M NaCl, 1M Tris, 0.5M EDTA, and 5% TritonX-100 at pH 8.0 with 4% protease inhibitor cocktail and 1% phenylmethylsulfonyl fluoride (PMSF; Sigma) (Cas # 329-98-6) for 20 min on ice. Tissue lysates were then centrifuged at 14,000 rpm for 15 min at 4°C and the supernatant was collected for protein estimation using bicinchoninic acid (BCA) assay (Thermofisher Scientific, Rockford, IL) (Verzella et al., 2020, 23225). Fifty micrograms of total protein were combined with loading dye at a 1:3 ratio. This was then loaded onto a 10% SDS polyacrylamide gel (Expedeon, Inc. San Diego) and then run for an hour at 150 V. Proteins were transferred from gel to Polyvinylidene fluoride (PVDF) membrane (Bio-Rad laboratories, Inc. Hercules, CA) (Cat #1620174) at 4°C at 100 V for 60 min in transfer buffer. Membrane was blocked using Pierce protein-free T-20 (TBS) blocking buffer (Thermofisher Scientific, Rockford, IL) (Cat #37571) for 30 min and incubated overnight at 4°C with pTAK1 on day 1 β tubulin antibody at 1:10,000 on day 2 as our endogenous control. Following treatment with primary antibodies, the membranes were washed with 1X TBST twice and subsequently incubated with a peroxidase conjugated secondary antibody (1:5000 goat anti-rabbit HRP in 1X TBST) (Invitrogen Cat #31460) at room temperature for 1 hour in the dark. The membranes were then treated with SuperSignal West Femto Chemiliminescent Substrate (Thermofisher Scientific, Rockford, IL) (Verzella et al., 2020, 34095) for 2 minutes and was covered in saran wrap and stored in a light protective cassette. The blot was visualized using x-ray imaging films (Thermoscientific, Rockford, IL). To determine if the detected band was specific, antibody was diluted to 0.2 μg/ml and half of the antibody solution was incubated overnight at 4°C with 1.0 μg/ml of immunizing peptide. E8 retinal samples run on SDS Page and transferred as indicated above were incubated with either normal antibody diluted to 0.2 µg or preabsorbed antibody pTAK1 antibody and signal detected as above.

### Ex ovo cultures/whole retinal explants

The method for retinal explants was adapted from (Shirazi Fard et al., 2015). Briefly, extraocular tissue was removed from enucleated eyes and eyes subsequently incubated in 37°C 1X PBS to aid in removal of pigmented epithelium with sharpened #5 Dumont forceps. Explants were transferred into a well containing 1 ml culture medium. Culture medium was prepared using 10% FBS, 2 mM Glutamax™, 10u/mL penicillin streptomycin, 5ug/ml Insulin, and 1:1 DMEM: F12 Nutrient mix (Thermofisher Scientific, Rickford, IL) (Verzella et al., 2020, 10565018). Each retinal explant was incubated in a single well of a 24 well plate at 37°C for 1 h on a rotator shaker inside a cell incubator with 5% CO_2_ at 50 rpm. Following this, retinal explants were either treated with vehicle (DMSO) as a control, or with 2 µM TAK1-specific inhibitor, (5Z)-7-Oxozeaenol (Tocris) (Cat #3604). In some experiments, ex ovo cultures were treated with a second TAK1 inhibitor known as Takinib at a concentration of 100p.m. (B2324; BioVision, Waltham, MA). Following treatment with vehicle or inhibitor, retinal explants were incubated at 37°C on a shaker with 5% CO_2_ at a speed of 50 rpm for 24 h. Subsequently, the 24 well plate containing the explants was removed from the incubator, retinal transplants were transferred into a new 24-well plate containing 4% paraformaldehyde in 0.1M phosphate buffer, pH 7.4 and incubated on ice for 15 min. Afterwards, retinal transplants were rinsed with 1X PBS for 10 min in the 24 well plate on ice. Whole retinal explants were transferred to wells with increasing concentrations of sucrose: 5%, 10%, 15%, and 20% sucrose in 1X PBS, incubating on ice for 1–2 h. Retinal explants were then placed into 3:1 OCT: sucrose solution and into an embedding mold. Embedded retinal explants were flash frozen on dry ice until frozen solid. Samples were stored at −80°C until ready to be sectioned.

### Quantitation of biomarkers, measurement of retinal width, and statistical analysis

Cryosections were double-labeled with antibodies specific for a given protein and Hoechst 33342, a UV fluorescent stain that labels all fixed nuclei stain, which labels all cell nuclei. Digital images of the central region of each retina (*N* = 3 retina for each vehicle and TAKi-treated retina) was taken using either an Olympus Fluoview FV 1000 or a Keyence semi-automated microscope. Each of the markers used in this study was quantitated by counting the number of cells immunolabeled in every 10th cryosection through the treated and control retinas. The number of immunolabeled cells was expressed as percentage of total number of cells labeled with Hoechst. Measurements of retinal width was determined in every 10th cryosections of E6 vehicle or TAKi-treated retinal cultures (*N* = 3 for each control and TAKi-treatments). Images of retinas were imported into ImageJ/FIJI using the measurement tool from the outer edge of the developing retina up to and including the nerve fiber layer. Students t-test was used to test for statistically significant differences between vehicle and TAKi-treated retinas. One-way ANOVAs were performed in some instances using GraphPad Prism version 9.0 for Windows with a Tukey post-hoc analysis (GraphPad Software, San Diego, CA).

## Results

### Phosphorylated TAK1 was found in all cells of the retina, but was more heavily expressed in actively dividing and differentiating cells of the developing chick retina

To examine the localization of TAK1 in the developing chick retina, immunohistochemistry was performed on sections from embryos at embryonic day 3 (E3), E5, E8, E15, and E18. E3 is representative of the stages when the retina is populated primarily with proliferative progenitors. E5 is representative of stages when early retinal cell types, such as cone photoreceptors and retinal ganglion cells are present, in addition to proliferative progenitors. E8 is a mid-developmental stage when most cell types have been generated in the retinal fundus and distinct outer and inner plexiform layers are beginning to form. By E15, there is a fully laminated retina; however, cells are still differentiating and refining connections. By E18, a mature retina is present, although there is evidence that some cell types continue to develop (Morrow et al., 1998).

**FIGURE 1:**
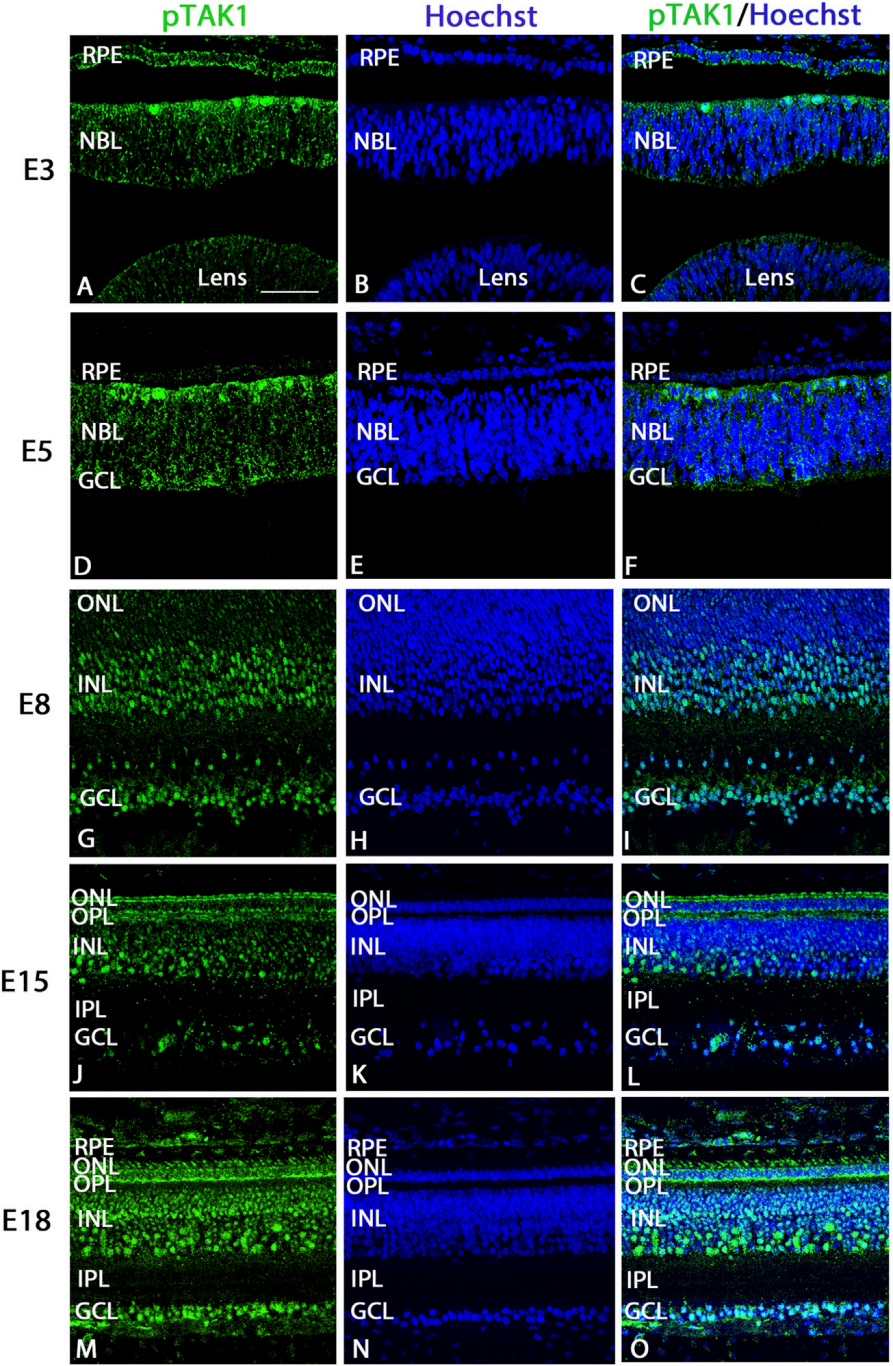
Phosphorylated TAK1 (pTAK1) weakly labels progenitor cells and heavily labels dividing and differentiating cells of the developing retina. Retinal sections from E3, E5, E8, E15, and E18 chick embryos were co-labeled for pTAK1 **(A,D,G,J,M)** and Hoechst nuclear stain **(B,E,H,K,N)**. Overlap in labels is shown in **(C,F,I,L,O)**. Abbreviations: RPE, retinal pigmented epithelium; NBL, neuroblast layer; INL, inner nuclear layer; GCL, ganglion cell layer; OPL, outer plexiform layer; IPL, inner plexiform layer. Scale bar in A = 50 μm for images **(A–O)**.

Retinal cryosections were labeled using an antibody that recognizes activated TAK1, phosphorylated at threonine 184/187 (pTAK1) along with a Hoechst label to visualize nuclei in the tissue. Overall, pTAK1 appeared to be localized throughout the retina, but was more heavily localized in actively dividing and differentiating cells. At E3, pTAK1 heavily labeled dividing cells at the scleral edge of the retina and weakly labeled retinal pigmented epithelium (RPE), retinal neuroblasts, and lens (Figures 1A–C). The RPE and cells of the retinal neuroblast layer (NBL) continued to be weakly labeled at E5 (Figures 1D–F); however, cells at the vitreal edge of the retina in the presumptive ganglion cell layer (GCL) appeared to be more strongly labeled (Figures 1D–F). Dividing cells at the scleral edge were still strongly labeled for pTAK1 (Figures 1D–F). At E8, the GCL and inner part of the inner nuclear layer (Wiley et al., 2005), where presumptive amacrine cells would be found, were strongly immunolabeled for pTAK1, whereas the cells of the outer nuclear layer and outer portion of the INL were either negative or weakly labeled (Figures 1G–I). Immunolabel at E15 appeared to be widespread in the retina, with strong signal present in the inner part INL, GCL, and outer plexiform layer (OPL), while weaker label was present in the remaining retina (Figure 1J–L). Label in the ONL was restricted primarily to the inner segment (Figure 1J–L). Finally, at E18, pTAK1 appeared to be localized throughout the retina, with similar patterns and strengths as those found at E15 (Figure 1M–O). In addition to the photoreceptor inner segments, signal also appeared to be present in the outer segment region (Figure 1M–O). Retinal localization patterns were confirmed by performing immunofluorescence using a second antibody that also recognizes pTAK1 (T184, 187) at E8 (Supplementary Figure S1).

Sections from each stage treated with isotype specific IgG in place of the primary antibody showed weak background label at the scleral edge of the retina at E5, E12-18 and weak label in the outer plexiform label at E12. Otherwise, the retina was negative for any immunofluorescence (Supplementary Figure S2A). Retinal lysates from E5, E8, E15 and E18 embryos were analyzed for pTAK1 expression by western blot (Supplementary Figure S2B). At each stage, a single band was detected at a molecular weight of approximately 68 kDa. Specificity of the pTAK1 antibody was tested by preabsorbing antibody with the immunizing peptide in western blots and sections (Supplementary Figures S2, S3). In sections of the retina, weak, non-specific label was detected primarily at the scleral edge of the retina from E3-E12, and in the outer plexiform layer at E15 and E18 (Supplementary Figure S3).

### pTAK1 labeled dividing cells strongly and retinal progenitors weakly

**FIGURE 2:**
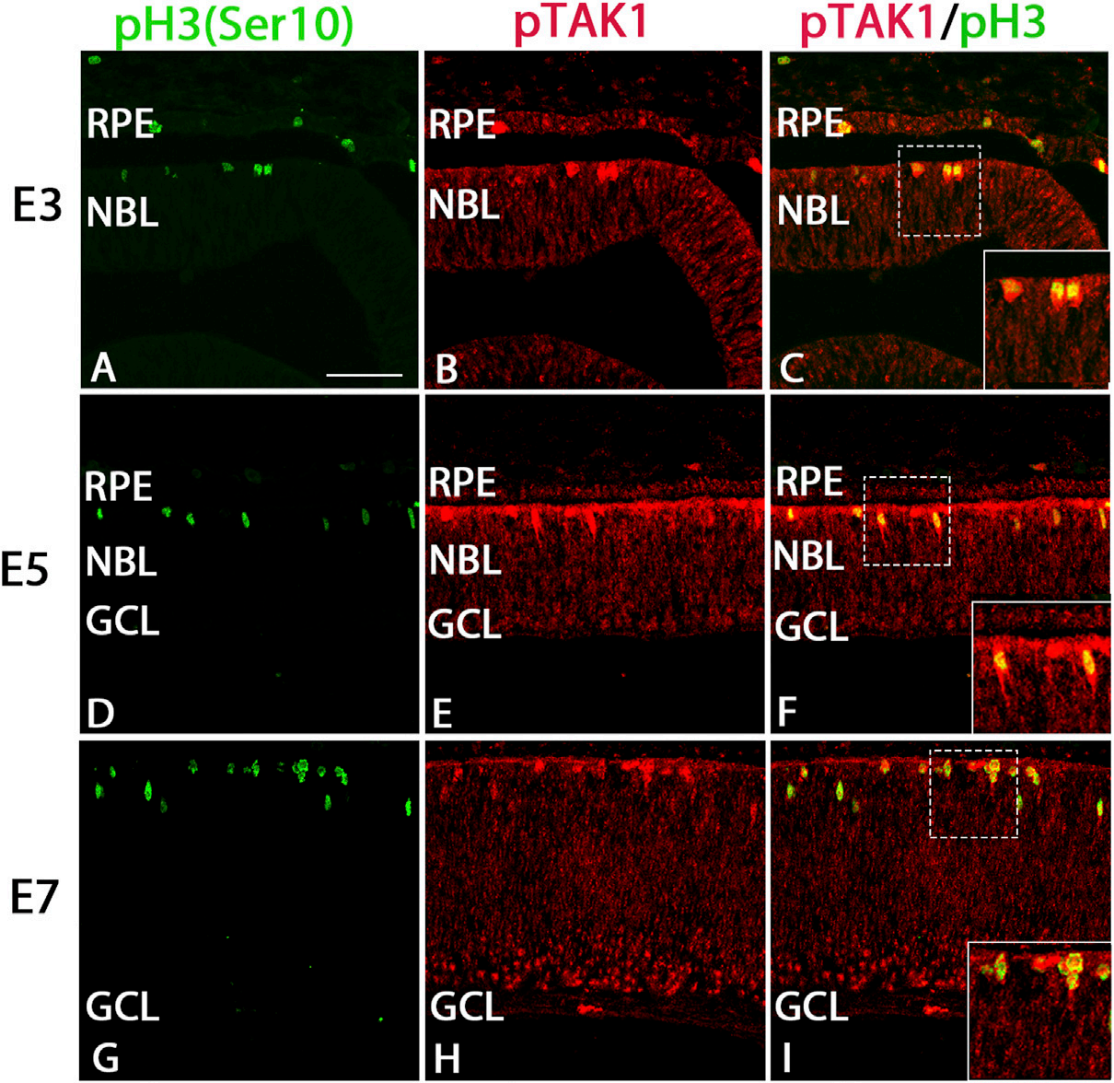
pTAK1 was co-localized to the phospho-histone 3^+^ (pH3^+^) dividing cells of the early chick retina. Chick retinal sections from E3, E5, and E7 embryos were double-labeled for pH3 **(A,D,G)** and pTAK1 **(B,E,H)**. Co-label is shown in **(C,F,I)**. Dashed boxes in **(C,F,I)** are shown at higher magnification in insets in **(C,F,I)**. Abbreviations: RPE, retinal pigmented epithelium; NBL, neuroblast layer; GCL, ganglion cell layer. Scale bar in A = 50 μm for images **(A–I)**.

**FIGURE 3:**
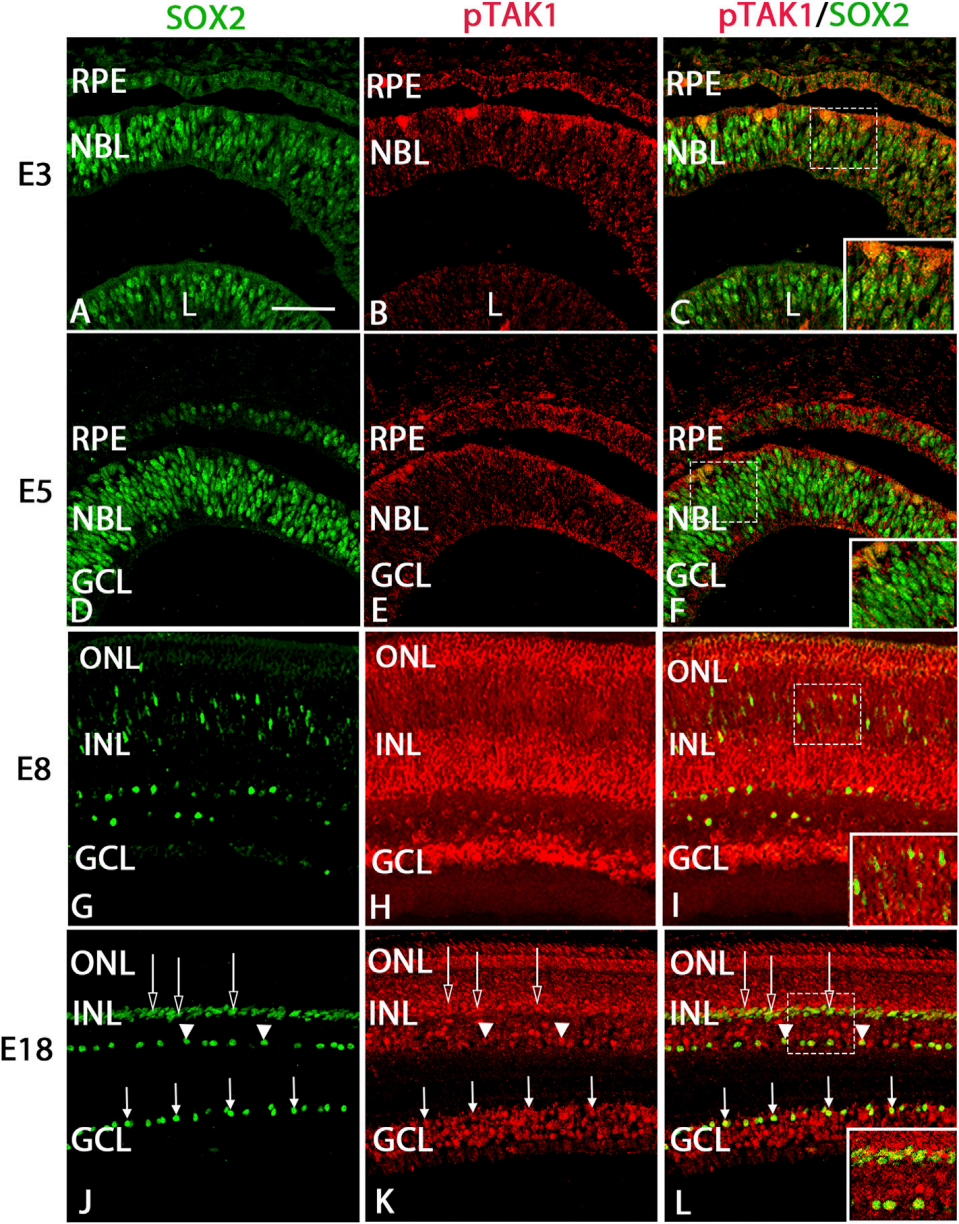
pTAK1 was co-localized to SOX2^+^ retinal progenitors in the early chick retina and a subset of Müller glial, cholinergic amacrine, and astrocyte cells in later stages.Chick retinal sections from E3, E5, E7, and E18 embryos were double labeled for SOX2 **(A,D,G,J)** and pTAK1 **(B,E,H,K)**. Co-label is shown in **(C,F,I,L)**. Open arrows indicated Müller glia, closed arrows indicate retinal astrocytes, and arrowheads indicate cholinergic amacrine cells **(J,K,L)**. Dashed boxes in **(C,F,I,L)** are shown at higher magnification in insets in **(C,F,I,L)**. Abbreviations: RPE, retinal pigmented epithelium; NBL, neuroblast layer; INL, inner nuclear layer; GCL, ganglion cell layer. Scale bar in A = 50 μm for images **(A–L)**.

Examination of immunolabel in Figure 1 suggested that pTAK1 was heavily localized to dividing retinal progenitor cells and lightly to non-dividing progenitors. To confirm pTAK1 localization in both progenitor populations, double-label fluorescence was carried out. Sections from E3, E5, and E7 double-labeled for phospho-histone 3 (pH3), a marker of prophase in proliferating cells, and pTAK1 were analyzed for co-label (Figures 2A–I). At each stage examined, pH3^+^ cells all appeared to be co-labeled with pTAK1. To determine if pTAK1 was also present in retinal progenitors not actively dividing, sections from E3, E5, E8, and E18 embryos were double-labeled for SRY (sex determining region Y)—box 2 (SOX2) and pTAK1. At early stages (E3 and E5), SOX2 labels all retinal progenitors, while in later stages (E8 and E18) SOX2 labels Mϋller glial cells, astrocytes, and cholinergic amacrine cells. E3 and E5 retinal sections co-labeled with SOX2 showed a high degree of overlap with pTAK1 (Figures 3A–I). At later stages of the retina, E8 and E18, SOX2 labeled cholinergic amacrine cells (Figure 3J–L, arrowheads), Müller glia (Figure 3J–L, open arrows) and retinal astrocytes (Figure 3J–L, closed arrows).

### pTAK1 was localized to populations of cells expressing cell type-specific markers at E8 and E18

**FIGURE 4:**
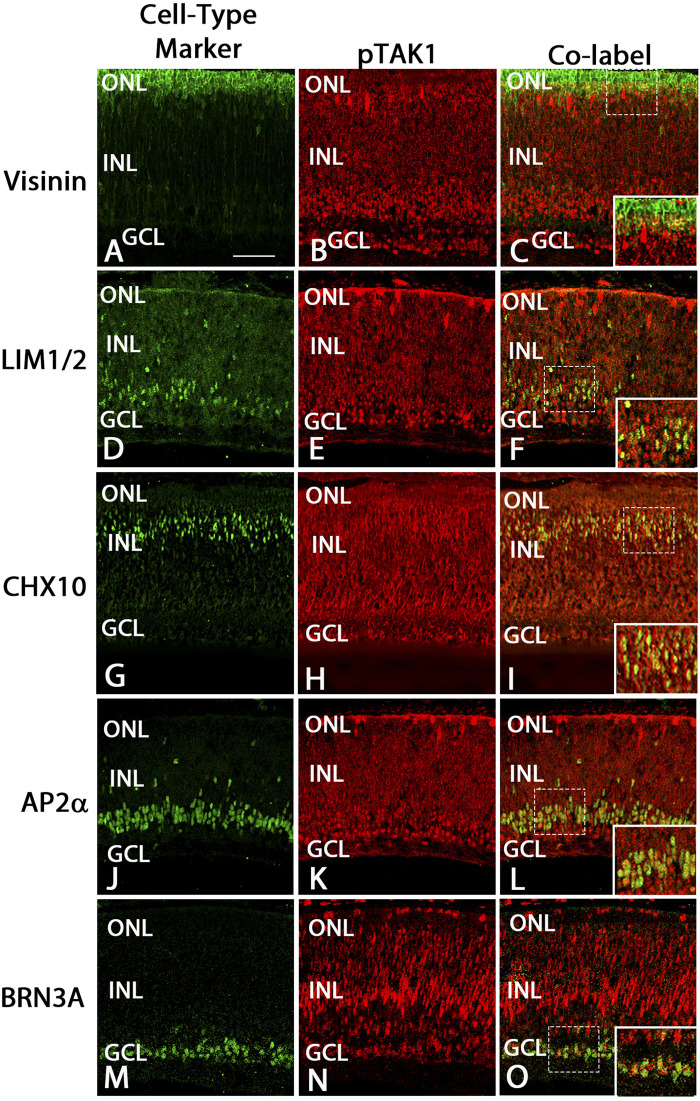
pTAK1 was localized to differentiating cells in E8 retina.Chick retinal sections from E8 embryos were double-labeled for pTAK1 and Visinin (cones; **A–C**), LIM1/2 [horizontal cells; **(D–F)**], CHX10 [bipolar cells; **(G–I)**], AP2α [amacrine cells; **(J–L)**] or BRN3A [ganglion cells; **(M–O)**]. Dashed boxes in **(C,F,I,L,O)** are shown at higher magnification in insets in **(C,F,I,L,O)**. Abbreviations: ONL, outer nuclear layer; NBL, neuroblast layer; INL, inner nuclear layer; GCL, ganglion cell layer. Scale bar in A = 50 μm for images **(A–O)**.

**FIGURE 5:**
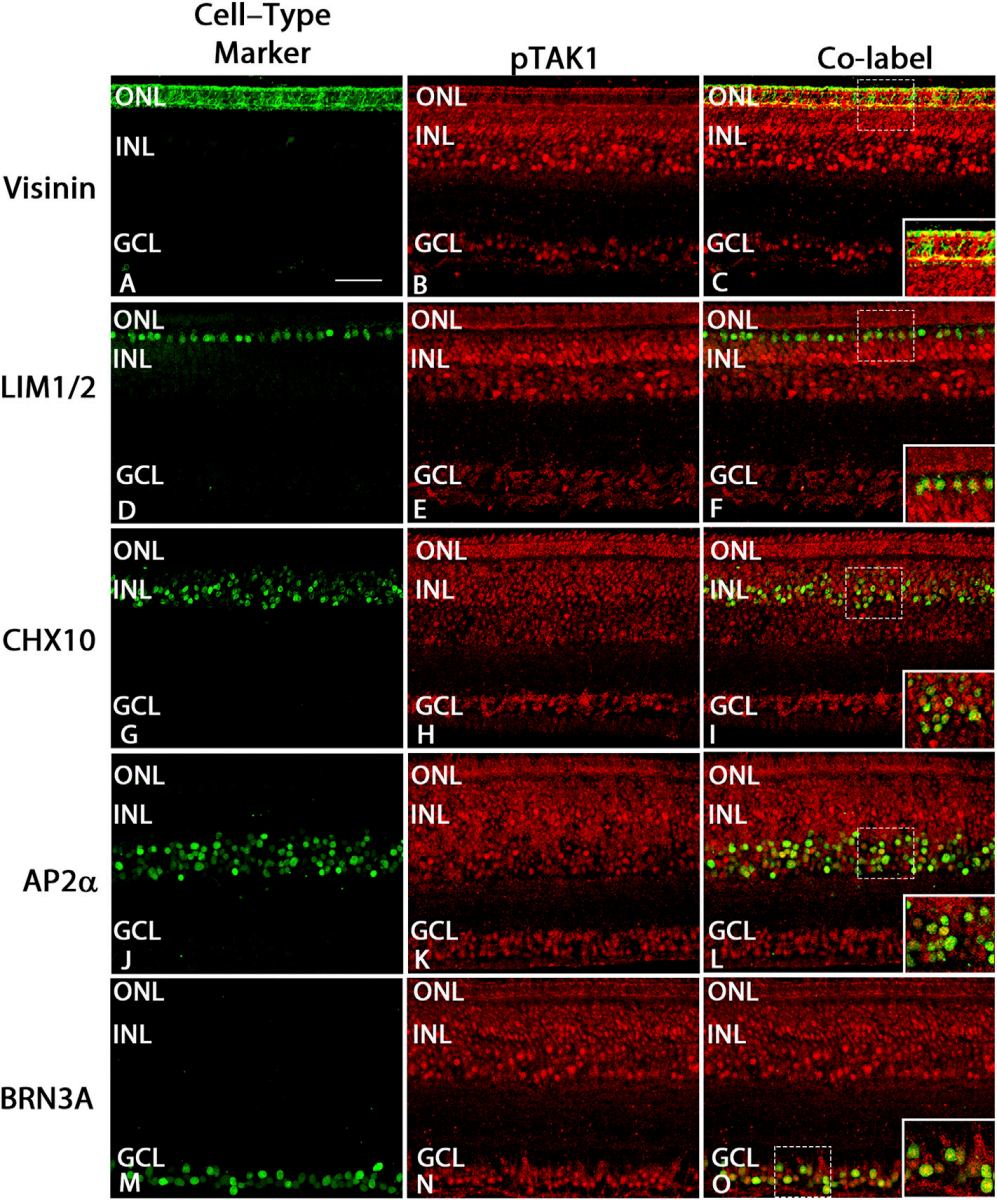
pTAK1 was localized to differentiated cells at E18. Chick retinal sections from E18 embryos were double-labeled for pTAK1 and Visinin [photoreceptors; **(A–C)**], LIM1/2 [horizontal cells; **(D–F)**], CHX10 [bipolar cells; **(G–I)**], AP2α [amacrine cells; **(J–L)**], or BRN3A [ganglion cells; **(M–O)**]. Areas shown by dashed boxes in **(C,F,I,L,O)** are enlarged and shown in insets in **(C,F,I,L,O)**. Abbreviations: ONL, outer layer; INL, inner nuclear layer; GCL, ganglion cell layer. Scale bar in A = 50 μm for images **(A–O)**.

To determine in which populations of differentiated cells pTAK1 might be found, we analyzed sections from E8 and E18 double-labeled for pTAK1 and cell type-specific markers such as SOX2 (cholinergic amacrine, Müller glia, and astrocyte cells), LIM1/2 (horizontal cells), CHX10 (bipolar cells), AP2α (amacrine), and BRN3A (ganglion cells). At E8, TAK1 was co-localized with cells positive for SOX2, LIM1/2, CHX10, AP2α, and BRN3A (Figures 3G–I and Figure 4). A subpopulation of cells labeled with Visinin appeared to be co-labeled with TAK1 (Figures 4A–C). TAK1 also co-localized with cells expressing SOX2, LIM1/2, CHX10, AP2α, and BRN3A at E18 (Figure 3J–L, Figure 5D–O). TAK1 also heavily labeled inner and outer segment regions and the synaptic region of Visinin^+^ photoreceptors at E18 and weakly labeled photoreceptor cell bodies (Figures 5A–C).

### Retinas treated with TAK1 inhibitor in ex ovo cultures show reduced number of phospho-JNK labeled cells (pJNK) and an increased number labeled with cleaved caspase 3

**FIGURE 6:**
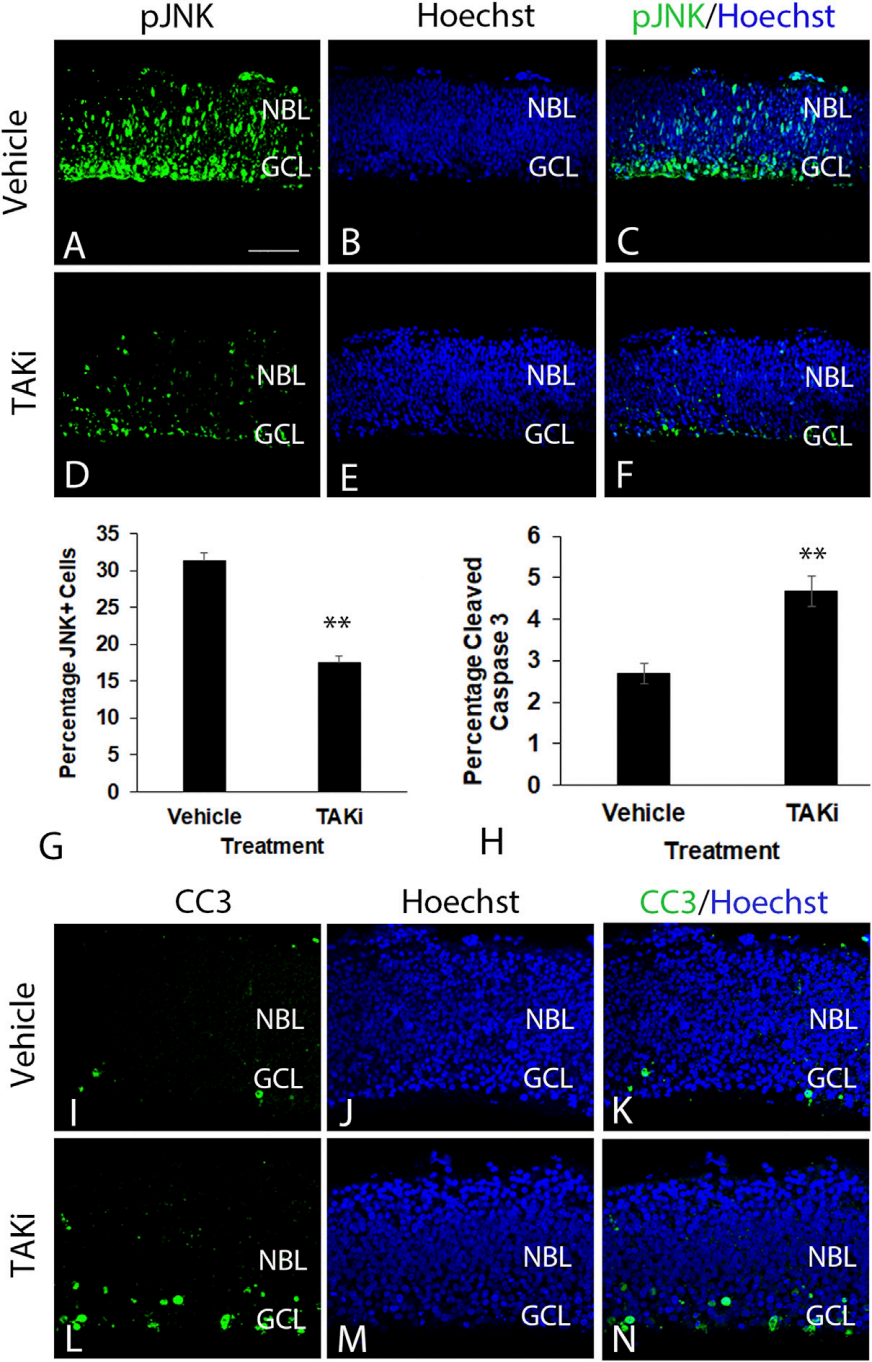
In comparison to vehicle-treated retinas, TAKi-treated retinas have a decrease in JNK labeling and an increase in cleaved-caspase 3 labeling. E6 chick retinal cultures were treated with vehicle or 2 μm TAK1 inhibitor (Sanjo et al., 2019) for 24 h and sections through retinas were co-labeled for downstream kinase phospho- c-jun N-terminal kinase [pJNK; **(A,C,D,F)**] or apoptosis marker cleaved caspase 3 [CC3; **(I,K,L,N)**] and Hoechst dye for DNA [blue; **(B,C,E,F,J,K,M,N)**]. Percentages of labeled cells were quantified in 3 separate retinas for pJNK+ cells **(G)** or cleaved caspase 3 **(H)**. Standard deviation is shown in **(G,H)**. A = 50 μm for images (**A–F** and **I–N**). Abbreviations: NBL, neuroblast layer; GCL, ganglion cell layer. Scale bar in A = 50 μm for images (**A–F** and **I–N**).

To examine the potential role of TAK1 in the differentiation of retinal cells, ex-ovo cultures of E6 retinas were treated with TAK1 inhibitor (Sanjo et al., 2019), 5Z-7-oxozeaenol. Biochemically, 5Z-7-oxozeaenol forms a covalent bond with TAK1 and inhibits the kinase activity as well as the ATPase activity (Wu et al., 2013). Initially, we determined whether the inhibitor was capable of reducing the phosphorylated and activated downstream target, c-jun n-terminal kinase (pJNK). E6 retinas were grown for 24 h in vehicle or TAKi, sectioned and immunolabeled for pJNK (Figure 6). Phospho-JNK labeled cells were widespread in vehicle-treated retinas (Figures 6A–C), while TAKi-treated retinas showed a marked reduction of label in comparison (Figures 6D–F). Quantitation of the number of pJNK-labeled cells in vehicle-treated retinas (*N* = 3) indicated 31.3% ( ± 3.52% SD) of the cells were pJNK^+^, whereas the percentage of pJNK^+^ cells in TAKi-treated retinas (*N* = 3) was 17.45% ( ± 2.03% SD) (Figure 6G).

Previous investigations in some tissues have shown TAK1 to be involved in cell death (Lamothe et al., 2013; Shin et al., 2013; Mihaly et al., 2014). To investigate the role of TAK1 in developing retinal cell death, we examined the activation of cell death marker caspase 3 (cleaved caspase 3; CC3) in vehicle- and TAKi-treated retinas (Figure 6I–N). In sections immunolabeled for CC3, there appeared to be an increase in CC3^+^ cells in sections from TAKi-treated retinas (Figures 6L–N) in comparison to vehicle-treated (Figures 6I–K). In both vehicle- and TAKi-treated retinas, CC3^+^ cells appeared to be localized to the GCL. Quantitation of sections through retinas (*N* = 3 for each treatment) indicated that vehicle-treated retinas had 2.70% ( ± 0.25% SD) cells were CC3^+^, while TAKi-treated retinas contained 4.67% ( ± 0.36% SD) (Figure 6H).

### Inhibition of TAK1 leads to an increase in the number of pH3- and SOX2-labeled cells

**FIGURE 7:**
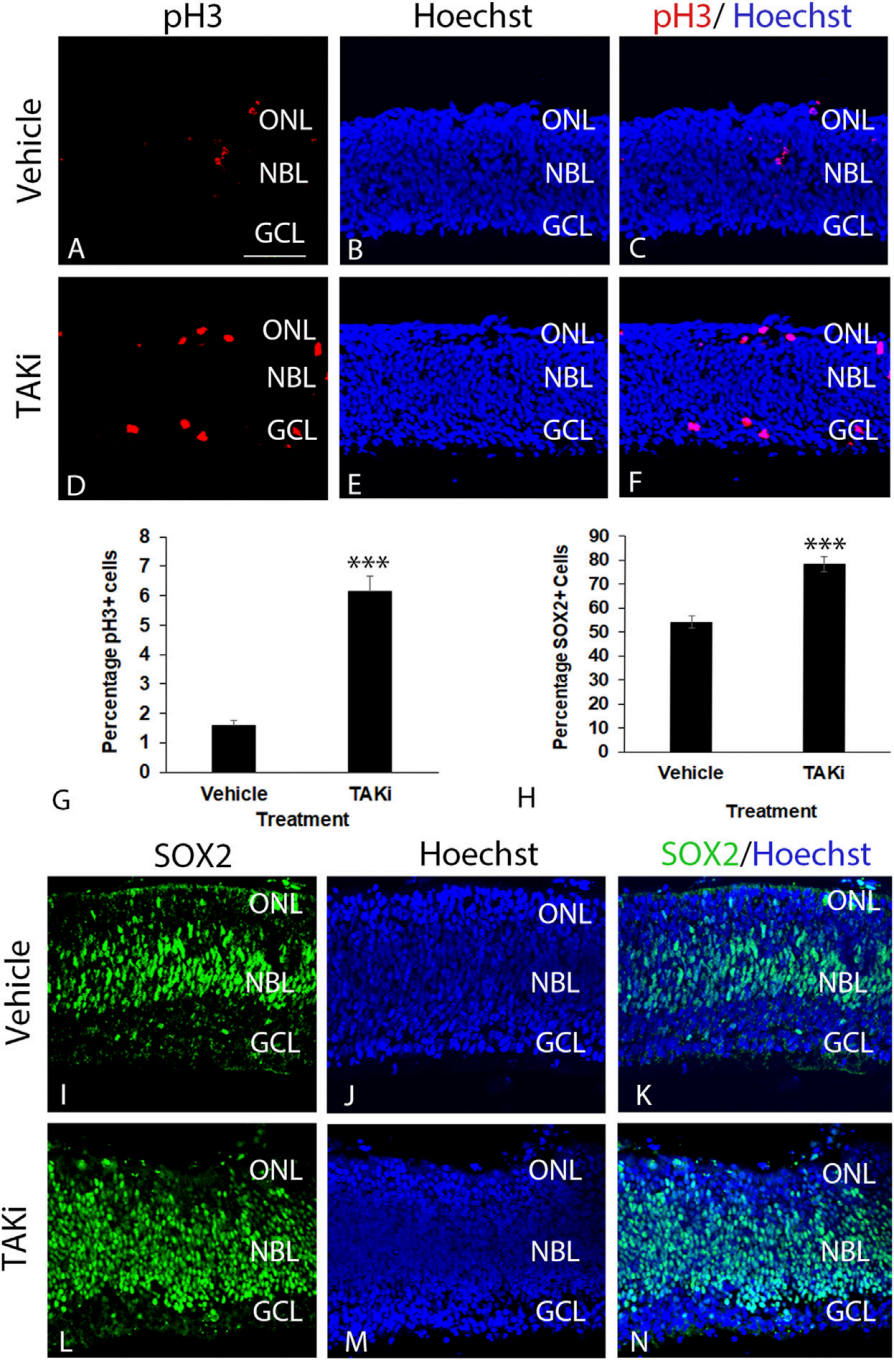
Taki-treated retinas have an increase in pH3+ and SOX2+ retinal progenitors in comparison to vehicle-treated retinas. E6 chick retinal cultures were treated with vehicle or 2μm TAK1 inhibitor (Sanjo et al., 2019) for 24 h and sections through retinas were co-labeled for cell division marker phospho-histone 3 [pH3; **(A,C,D,F)**] or progenitor marker SOX2 **(I,K,L[N)** and Hoechst dye for DNA [blue; **(B,C,E,F,J,K,M,N)**]. Percentages of labeled cells were quantified in 3 separate retinas for pH3+ cells **(G)** or SOX2+ cells **(H)**. Standard deviation is shown in **(G,H)**. A = 50 μm for images (**A–F** and **I–N**). Abbreviations: ONL, outer nuclear layer; NBL, neuroblast layer; GCL, ganglion cell layer. Scale bar in A = 50 μm for images **A–F** and **I–N**.

The localization of pTAK1 in dividing and differentiating cells led to the hypothesis that TAK1 was important in cell type-specific differentiation. If TAK1 was involved in cell cycle exit, the number of proliferating and/or retinal progenitor cells should increase in the presence of an inhibitor. To investigate whether there was a change in the number of dividing cells, retinas treated with vehicle or TAKi were sectioned and immunolabeled for pH3 (Ser10), a modification that is highly correlated with chromosomal condensation occurring in mitosis and meiosis (Van Hooser et al., 1998). Sections through the vehicle-treated retinas showed modest labeling for pH3 near the edge of the scleral edge of the retina (Figures 7A–C). In comparison, sections through TAKi-treated retinas showed a clear increase in the number of pH3^+^ cells at the scleral edge, and it was apparent that some aberrantly labeled cells localized within to the GCL were also present (Figures 7D–F). Quantification of vehicle-treated retinas indicated 1.58% ( ± 0.19% SD) were pH3^+^, while TAKi-treated contained 6.15% ( ± 0.52% SD) pH3^+^ cells (Figure 7G).

Localization of retinal progenitor cells was carried out localizing and quantitating cells positive for progenitor marker SOX2 in vehicle and TAKi-treated ex-ovo retinas (Figure 7H–N). In vehicle-treated retinas, SOX2 was abundant in the elongated nuclei of retinal progenitors in the neuroblast layer as well as in dividing cells at the scleral edge (Figures 7I–K). SOX2 also labeled cells primarily in the neuroblast layer of the TAKI-treated retina; however, the number of labeled nuclei appeared much greater than the vehicle-treated retinas and the labeled nuclei are round in appearance rather than the normal elongated nuclei (Figure 7L–N). Quantitation of SOX2-labeled cells upheld the observation that there were more labeled cells in the TAKi-treated retinas (78.24% ± 3.09% SD) in comparison to vehicle-treated retinas (54.10% ± 2.42% SD) (Figure 7H).

As an alternate means of determining whether there was an increase in proliferation in retinas TAKi-treated retinas in comparison to those treated with vehicle, the width of sections were measured from the outer region where cells undergo division to the inner region where the developing retinal nerve fiber layer is localized. The mean thickness of vehicle-treated retinas was 126.8 ( ± 25.8), while the mean thickness of the TAKi-treated retinas was 192.6 ( ± 52.2) (Supplementary Figure S5A). A *t*-test indicates a statistically significant difference between the two means (*p* < 0.0001). Moreover, when the number of sections that measured in 50 µm increments from 0 to 300 µm was counted, it became clear that the majority of sections measured between 150 and 300 µm whereas a large majority of vehicle-treated sections measured between50-150 µm (Supplementary Figure S5B). Last, each measurement graphed from the highest to lowest shows a clear increase in the size of TAKi-treated retinas in comparison to vehicle-treated retinas (Supplementary Figure S5C).

### Inhibition of TAK1 also leads to an increased number of cells labeled with visinin, islet1, NAPA73, and Brn3a

**FIGURE 8:**
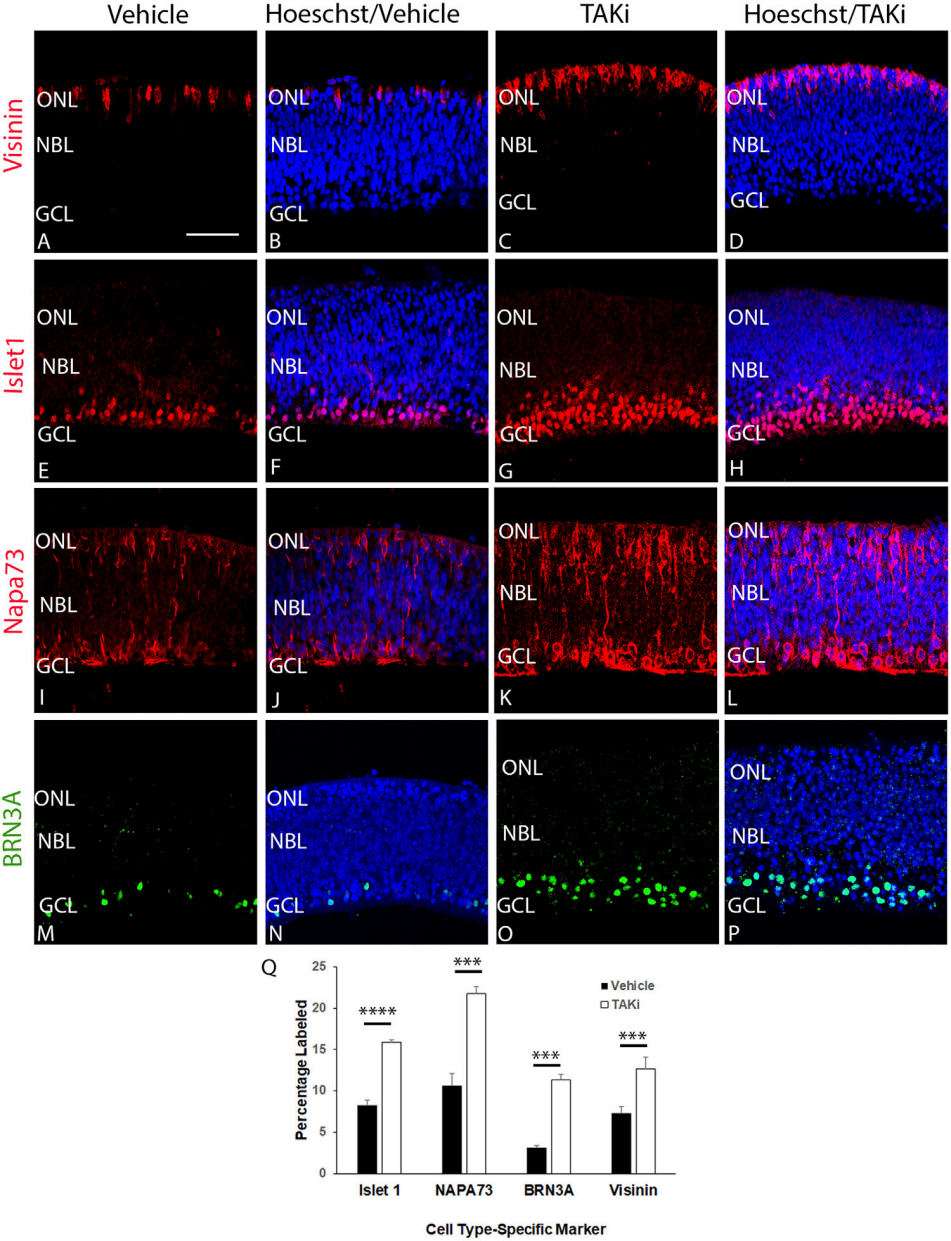
Sections through TAKi-treated retinas show an increased percentage of cells expressing cell type-specific markers Visinin, Islet1, NAPA73, and BRN3A in comparison to vehicle-treated retinas. E6 chick retinal cultures were treated with vehicle or 2 μM TAK1 inhibitor (Sanjo et al., 2019) for 24 h and sections through retinas were co-labeled with Hoechst dye (DNA; blue) and visinin **(A–D)**, islet1 **(E–H)**, napa73 **(I–L)**, or BRN3A **(M–P)**. Abbreviations: NBL, neuroblast layer; GCL, ganglion cell layer. Scale bar in A = 50 μm for images **(A–P)**. Quantitation of the percentage of labeled cells in vehicle and TAKi-treated retinas is shown in **(Q)**.

To determine if there was a change in the number of cells expressing differentiation-specific markers, we treated ex-ovo retinal cultures with vehicle or TAKi, localized and quantitated cells expressing visinin, islet1, NAPA73, and BRN3A (Figure 8). The ex-ovo cultures could not be cultured for long periods of time, hence the analysis was focused primarily on retinal cells that differentiate early, such as cones (Visinin) and ganglion cells (NAPA73 and BRN3A). The number of Visinin + cells in vehicle-treated retina averaged was 7.29 cells per section ( ± 0.82, *N* = 4 retinas), whereas TAKi-treated cells per section averaged 12.68 ( ± 1.46, *N* = 3 retinas). Likewise, immunolabel for NAPA73 in vehicle-treated retinas showed an average of 10.91 cells per section ( ± 1.43, *N* = 4 retinas) and TAKi-treated samples showed an average of 21.80 cells per section ( ± 0.84, *N* = 4). Brn3a + cells showed a similar increase, with vehicle-treated retinas showing 3.15 cells per section ( ± 0.021, *N* = 4) and TAKi-treated an average of 11.30 per section ( ± 0.74, *N* = 4). Islet1, a marker that has been shown to label cells undergoing differentiation, labels primarily ganglion cells at early stages, but later is found in most cells of the retina (Gutierrez et al., 2011). For this study, we used islet1 as a gauge of differentiation within the retina. Vehicle-treated retinas contained an average of 8.25 ( ± 0.59, *N* = 4 retinas) labeled cells per section. In comparison, TAKi-treated retinas contained an average of 15.92 ( ± 0.23, *N* = 4 retinas). There were clearly more cells expressing markers found in early differentiating retinal cell types within the TAKi-treated retinas in comparison to vehicle-treated retinas.

To increase confidence that TAK1 inhibitor 5Z-7-oxozeaenol was specifically affecting TAK1 activity, experiments quantitating cleaved caspase 3, pJNK, SOX2, Islet1, and Visinin were repeated using the highly selective and potent TAK1 inhibitor Takinib (Supplementary Figure S4). Results obtained with Takinib were similar to those obtained with 5Z-7-oxozeaenol, with an increase in both progenitor and differentiating populations of cells in the developing chick retina.

## Discussion

The present study focused on the role of TAK1 in the differentiation of cells in the developing chick retina. Immunolocalization of pTAK1 with markers present in progenitor and differentiating retinal cells indicated that pTAK1 was widely expressed in progenitor (dividing and non-dividing) and differentiating cells of the retina, although expression in dividing progenitors and differentiating cell populations was heavier than expression noted in progenitors that were not actively dividing. *Ex vivo* E6 retinal cultures treated with TAK1 inhibitors, 5Z-7-oxozeaenol or Takinib, for 24 h showed an increase in 1) progenitor cells (both actively undergoing mitosis and those that were not actively dividing), 2) apoptotic cells, and 3) cones and ganglion cells.

### TAK1 in cell cycle regulation

Prior to conducting this study, we hypothesized that TAK1 played a role in differentiation, as has been suggested by studies in other tissues (Ninomiya-Tsuji et al., 1999; Blonska et al., 2005; Lamothe et al., 2013; Mihaly et al., 2014). However, results using TAK1 inhibition in conjunction with *ex-ovo* retinal cultures indicated there were increases in cell type-specific differentiation, proliferating progenitors, and (albeit small) in apoptosis. Together, these results suggest that TAK1 may not be limited to a role in differentiation but may play a broader role in the cell cycle. TAK1 has some revealing links to the cell cycle. TAK1 has been shown to directly affect levels of both cyclins D1 and A, although none have been shown in the central nervous system (Terada et al., 1999a; Terada et al., 1999b). A constitutively active form of TAK1 expressed in kidney cells reduces ^3^H uptake by cells as well as passage through G2 to M phases. Further investigation indicated the constitutively active form of TAK1 inhibited both cyclin D1 and cyclin A promoter activity and the expression of protein (Terada et al., 1999a). Cyclin D1 is critical for progression of cells through the G1 phase of the cell cycle, while cyclin A is important in both synthesis and mitosis (Michele Pagano et al., 1992; Terada et al., 1999a). This finding is indicative of TAK1 inhibiting progression though G1 of the cell cycle, consistent with our observations that the inhibition of TAK1 in the retina leads to an increase in cell cycle progression and an increase in synthesis and G2 to M transition, leading to an increase in both proliferating and differentiating cells. TAK1 has also been implicated in reducing cell cycle progression following DNA damage in cells (Liaw and Chiang 2019). Inactive tousled-like kinase (TLK) bound to TAK1 increased p38 kinase activity, leading to an increased G2 phase. Collectively, these studies suggest that TAK1 may be playing a key role in the cell cycle. During normal development, TAK1 may reduce the activity of cyclin D1, allowing the cycle to lengthen in G1, and increasing the number of cells that leave the cell cycle as factors necessary for differentiation of later-born cell types accumulates. When TAK1 activity is decreased, there is less disruption to cyclin D1 activity, allowing cells to progress through G1 and the rest of the cell cycle, leading to an increase in cells that proliferate and an increase in the early-born cells of the retina. However, G1 phase beyond this threshold is what is necessary to drive differentiation, along with bHLH (Hardwick et al., 2015). Transcription factors of the bHLH proneural family are considered the master regulators of neurogenesis and are responsible for controlling progenitor maintenance through the Notch-Delta pathway, cell cycle exit, and differentiation (Hardwick et al., 2015). If cell cycle length determines whether cells can differentiate into early-born cell types versus later-born cell types, then we would predict that with the short G1 phase there should be an increase in early-born cell types and a reduction in later-born cell types; whereas if G1 length is increased earlier we would see a decrease in early-born cell types and an increase in later-born cells. Unfortunately, the *ex ovo* retinal cultures can only be performed for a limited time, so future experiments will focus on addressing these questions using other methods and model systems.

### TAK1 in apoptosis

TAK1 has been implicated in survival, apoptosis and necroptosis, depending on extracellular and intracellular context (Mihaly et al., 2014; Verzella et al., 2020). Survival appears to involve transcription of genes downstream of nuclear factor kappa-light-chain-enhancer of activated B (NFκB) and c-jun-N-terminal kinase (JNK). Studies using a dominant negative form of TAK1 in liver have shown that quiescent cells re-enter the cell cycle via expression of c-Myc (Schnabl et al., 2001). While c-Myc can drive cells into division it has also been shown to induce apoptosis. Cell death induced by c-Myc has been shown to be connected to the activation of apoptotic factors caspase-3 and c-Jun N-terminal kinases (JNKs) (Dhanasekaran and Reddy 2008). Whether the activation of NFkB leads to survival, apoptosis, or necroptosis is likely to depend on various present factors. RIPK1 and RIPK3 factors lead to necroptosis, JNK apoptosis, ATG5 and Beclin1 in autophagy (Verzella et al., 2020).

Multiple studies have shown that c-Myc is necessary for apoptosis in response to pathologies such as cancers, DNA damage, and chemotoxins (Prendergast 1999). In the central nervous system, a study found that in microglia, activated TAK1 resulted in an increase in pro-inflammatory cytokines and apoptosis (Zeyen et al., 2020). TAK1 inhibition in cancer cells *in vitro* has shown an increase in apoptosis through the inhibition of TNF survival and inflammatory signaling (Scarneo et al., 2020). TAK1 has also been found to regulate cell survival through oxidative stress and receptor-interacting protein kinase 1 (RIPK1) and when TAK1 is inhibited or knocked out, it results in apoptosis in most tissue types (Mihaly et al., 2014). TAK1 inhibition or dysregulation results in abnormalities that could be key to learning more about human pathologies.

### TAK1 in maintenance of the retina and in pathology

In these studies, we have shown that TAK1 is more widespread within cell types of the retina than previously observed. Collectively, the results of this study lead to the suggestion that TAK1 plays a dual role within the developing chick retina in dividing and differentiating cells. Previous work has shown that TAK1 expression that is found in the chick retina is similar to the TAK1 expression found in the human retina (Dvashi et al., 2016). Activated TAK1 has been found to be present in all layers of a normal retina while it is barely detectable in pathological retinas (Dvashi et al., 2016). This similarity between TAK1 prevalence in chick and human retinas sheds lights on possible approaches for studying human retinal pathologies. TAK1 has been implicated and shown to be involved in cell proliferation, cell death, and differentiation. Future studies exploring TAK1’s role in the cell cycle, proliferation, and cell death could elucidate potential therapies for retinal abnormalities and diseases.

### Future studies

The focus of this study was to investigate the potential role(s) that TAK1 plays in the differentiating retina. We found widespread localization of pTAK1 in both proliferating and differentiation cells in the chick retina and treatment of the E6 retina with a TAK1 inhibitors indicated that TAK1 plays a role in proliferation and differentiation of early born retinal cells. *Ex ovo* retinal culture, while useful, is limited in the amount of time retinas can remain in culture. Thus, a fuller investigation of TAK1 in retinal cells born later in development will be important to discerning whether the effect we see early in retinal development can be expanded to later developmental events. Likewise, investigation into TAK1 in other systems, such as a mammalian system will also be important as we expand our observations and use other tools to investigate TAK1 and the mechanisms of its actions in the retina.

The novel observation that inhibition of TAK1 increases both differentiation of early born retinal cell types as well as proliferating cells together with the knowledge that TAK1 is a hub kinase that is downstream of multiple pathways has led us to hypothesize that perhaps TAK1 is playing a role that common amongst all the upstream factors. In regards to the TGF-β superfamily, it may be important to activate both the canonical and non-canonical signaling pathways. To obtain cell type-specific differentiation in addition to removal of cells from the cell cycle. Thus future studies will focus on teasing apart the role(s) of canonical and non-canonical pathways in the retina (Table 1).

**TABLE 1:** Antibodies used in study.

Antigen	Source	Antibody dilution	RRID number
Phospho-TAK1 (Cat # ab79583)	Abcam (Cambridge MA)	1:100	AB_1659038
Phospho-TAK1 (cat# ab192443)	Abcam (Cambridge, MA)	1:100	AB_2819208
Phospho-histone 3 (Cat # 9706)	Cell Signaling (Danvers, MA)	1:800	AB_331748
AP2α (3B5)	Developmental Studies Hybridoma Bank (Iowa City, IA)	1:30	AB_528084
BRN3A (Clone5A3.2)	Millipore	1:100	AB_92154
SOX2 (Cat# sc17320)	Santa Cruz (Santa Cruz, CA)	1:250	AB_2286684
Visinin (7G4)	Developmental Studies Hybridoma Bank (Iowa City, IA)	1:30	AB_528510
Islet1 (39.4D5)	Developmental Studies Hybridoma Bank (Iowa City, IA)	1:30	AB_2314683
NAPA73 (E/C8)	Developmental Studies Hybridoma Bank (Iowa City, IA)	1:20	AB_531792
Phospho-JNK	Millipore	1:300	AB_310412
Cleaved caspase 3 (cat#9661)	Cell Signaling (Danvers, MA)	1:300	AB_2341188
β-tubulin (Cat# T0198)	SIGMA (St. Louis, MO)	N/A	AB_477556

## Data Availability

The original contributions presented in the study are included in the article/Supplementary Material, further inquiries can be directed to the corresponding author.
