# Supplementary figures and images for: TAK1 inhibition increases proliferation and differentiation of chick retinal cells
Source: Front Cell Dev Biol. 2022 Sep 13;10:698233. doi: 10.3389/fcell.2022.698233 (PMC9513612; doi:10.3389/fcell.2022.698233)

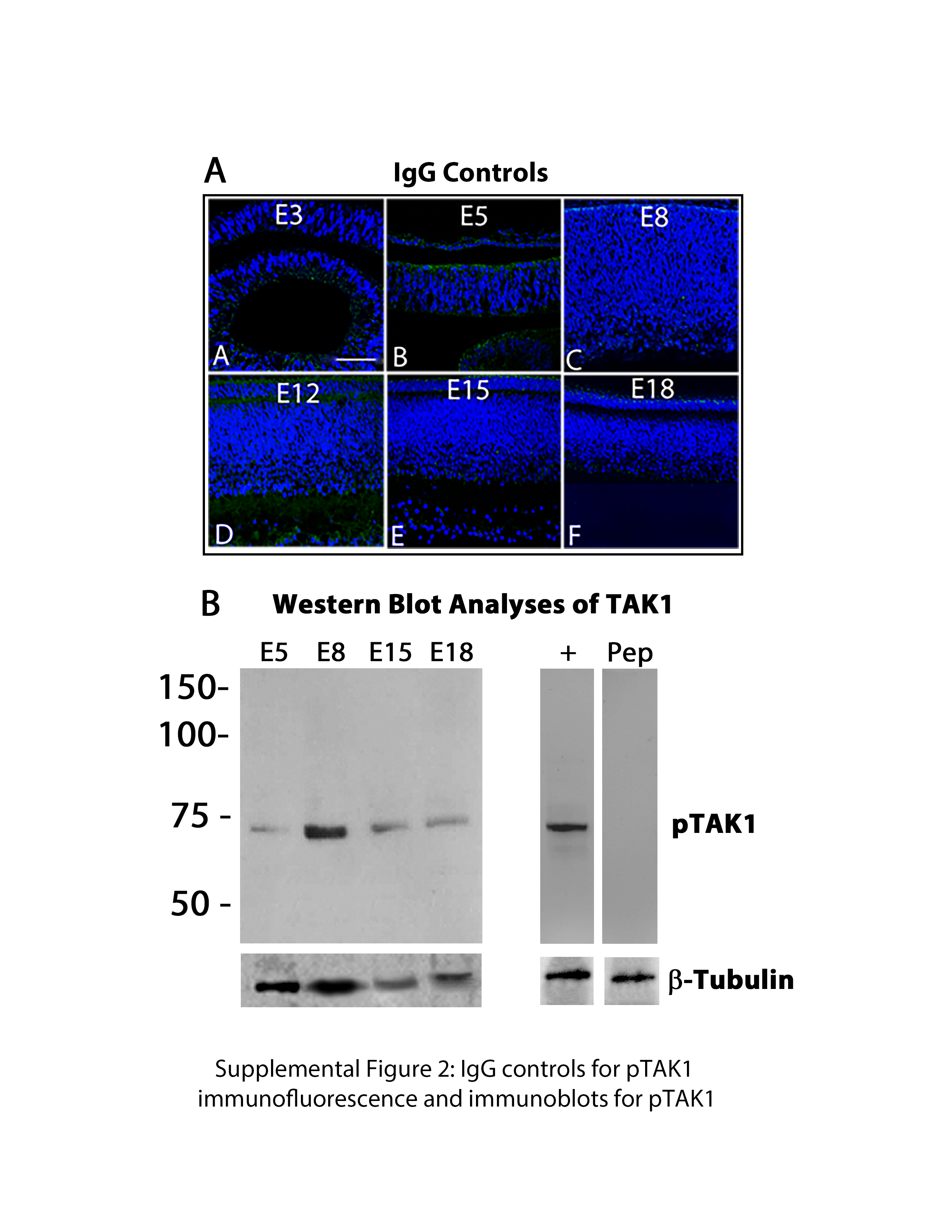

Supplement: Supplementary file 1 [file Image3.TIF]

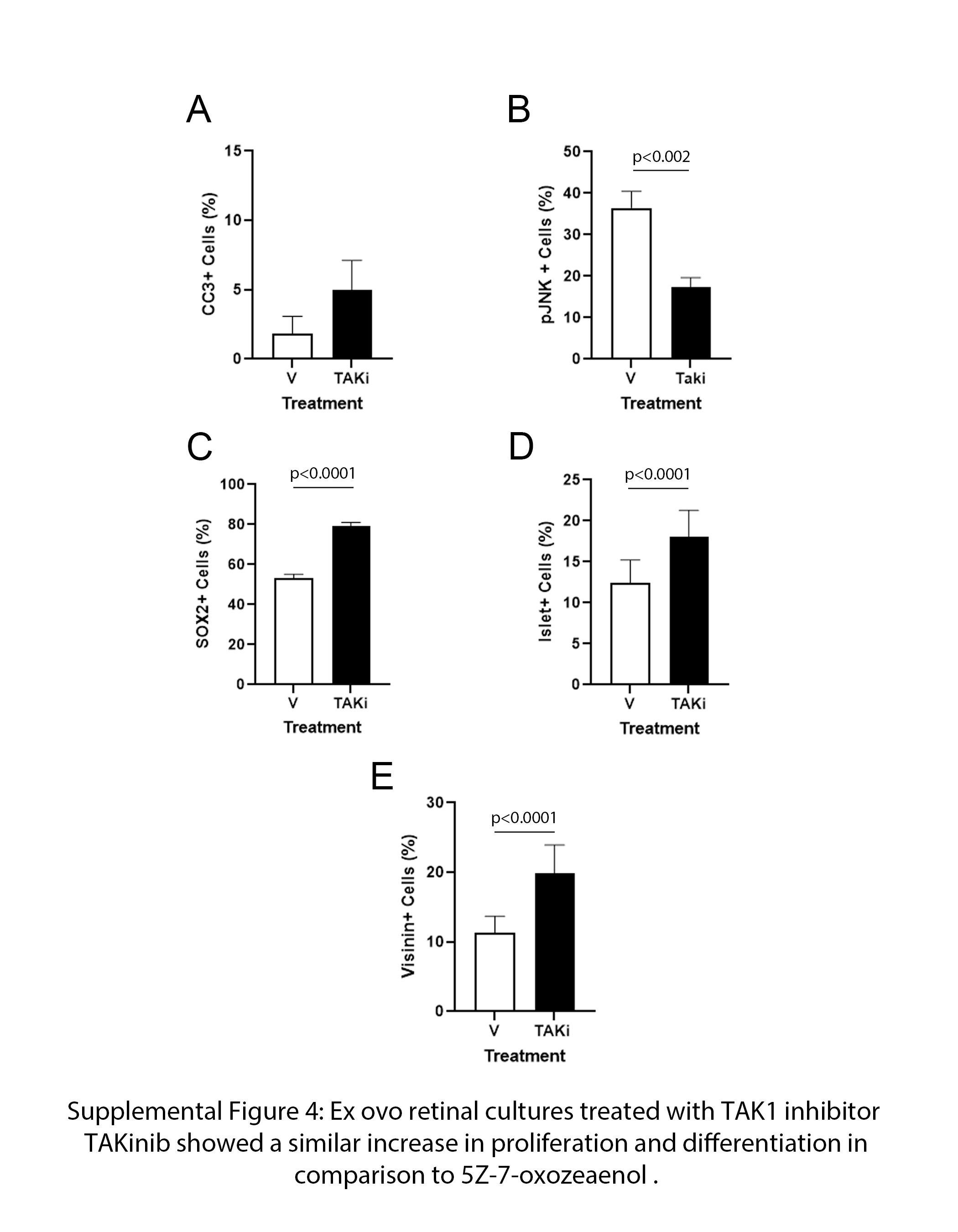

Supplement: Supplementary file 2 [file Image4.TIF]

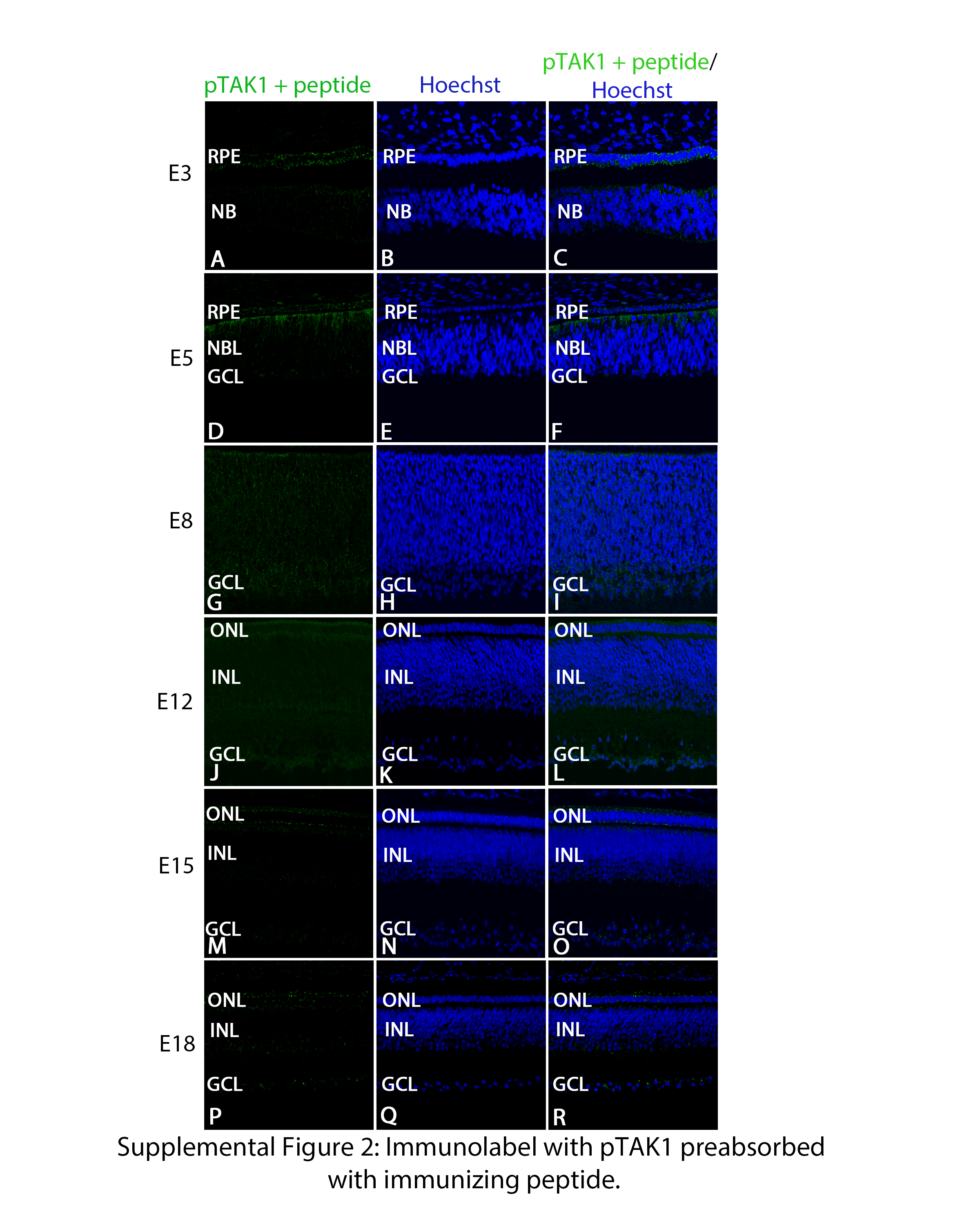

Supplement: Supplementary file 3 [file Image2.TIF]

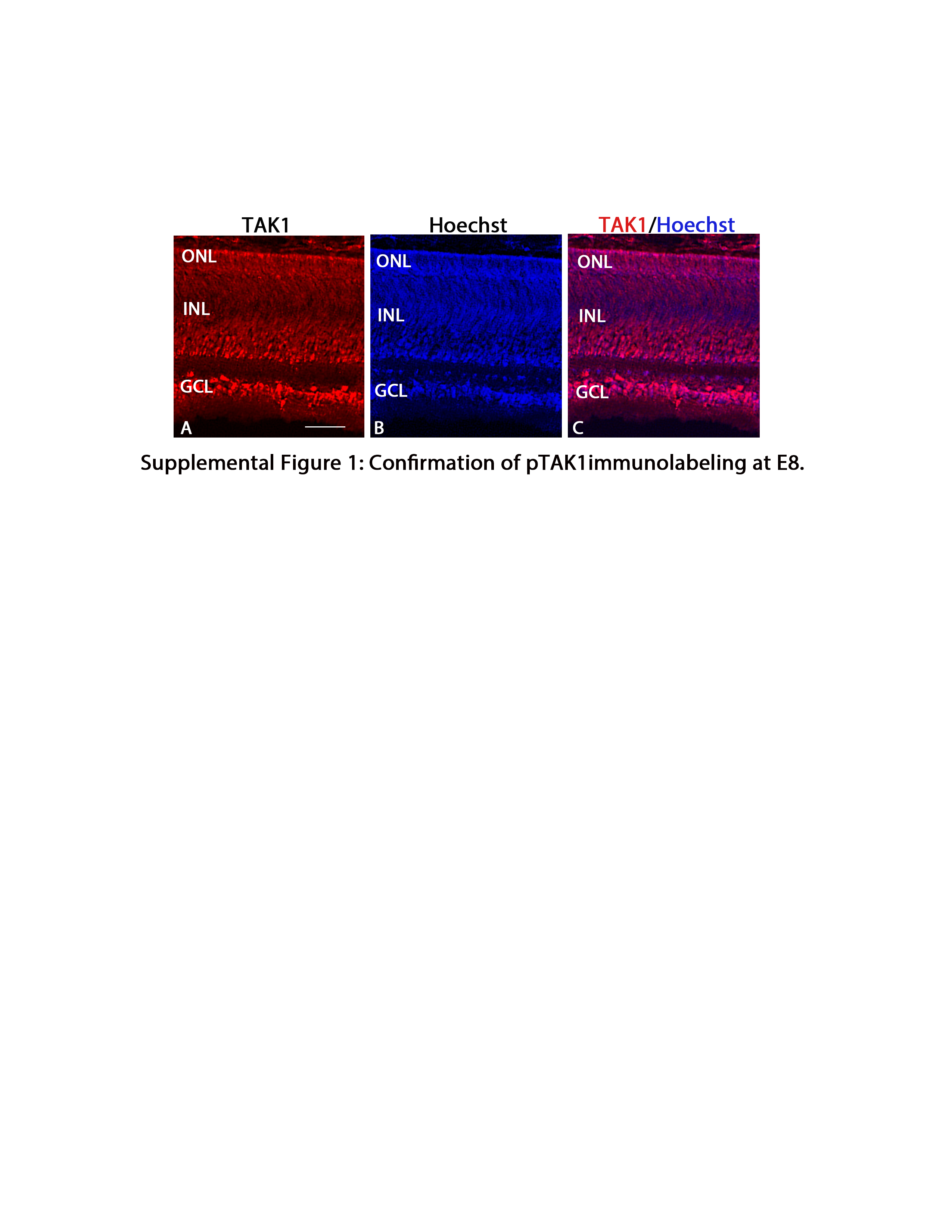

Supplement: Supplementary file 4 [file Image1.TIF]

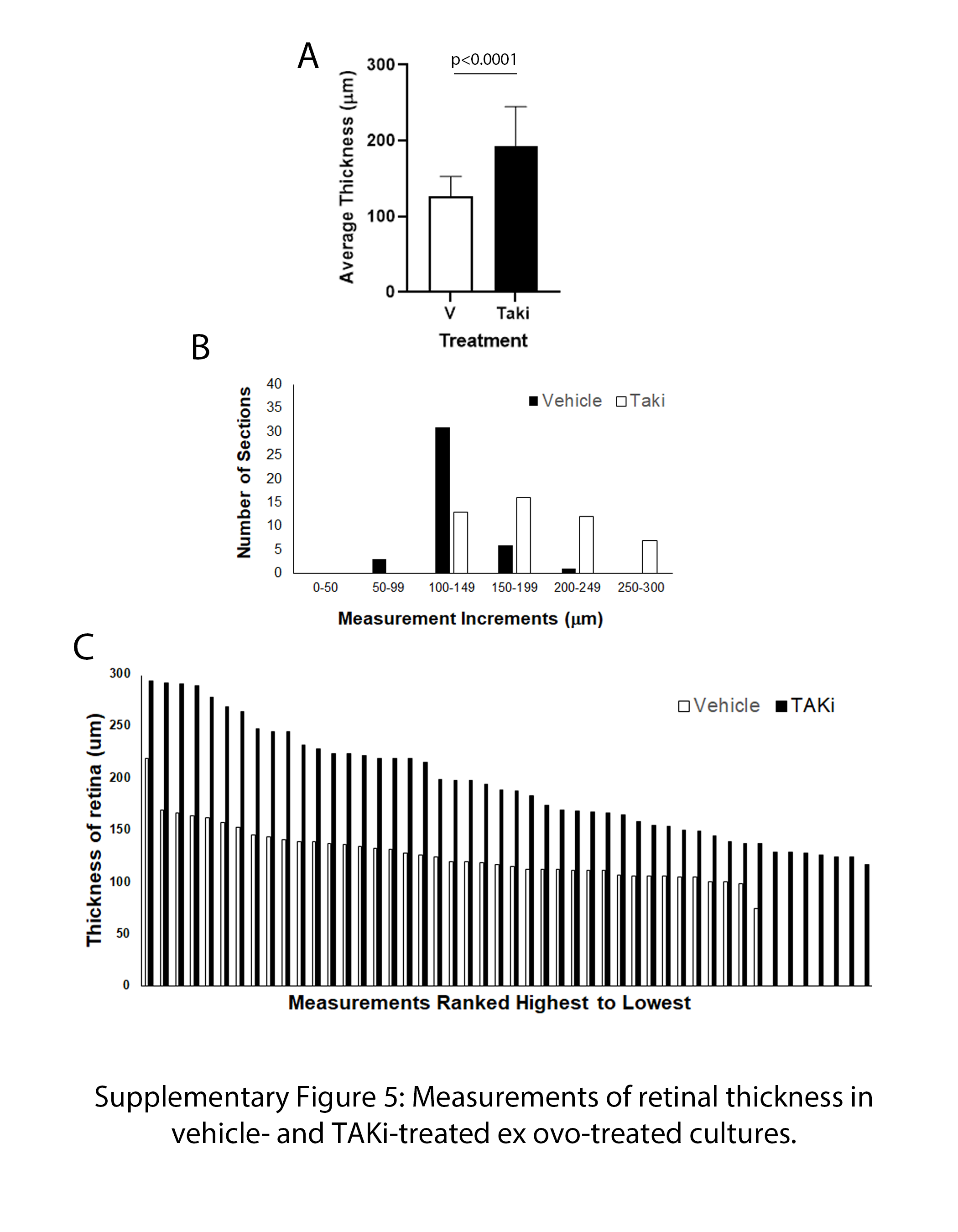

Supplement: Supplementary file 5 [file Image5.TIF]
