# Supplementary material for: TAK1 inhibition increases proliferation and differentiation of chick retinal cells
Source: Front Cell Dev Biol. 2022 Sep 13;10:698233. doi: 10.3389/fcell.2022.698233 (PMC9513612; doi:10.3389/fcell.2022.698233)
Supplement: Supplementary file 6 [file DataSheet1.docx]

**Supplementary Figure Legends**

**Supplemental Figure 1: Confirmation of pTAK1 localization.**

Retinal sections from E3, E5, E8, E15, and E18 chick embryos were co-labeled for pTAK1 using another primary antibody recognizing pTAK1 (A, D, G, J, M) and Hoechst nuclear stain (B, E, H, K, N). Overlap in labels is shown in C, F, I, L, O. Abbreviations: RPE, retinal pigmented epithelium; NBL, neuroblast layer; INL, inner nuclear layer; GCL, ganglion cell layer. Scale bar in A=50μm for images A-R

**Supplemental Figure 2: IgG controls for pTAK1 immunofluorescence and immunoblots for pTAK1.**

1. Retinal sections from E3, E5, E8, E12, E15, and E18 chick embryos were incubated with isotype-specific IgG in place of the primary antibody and incubated with secondary as usual. B) Immunoblot of lysates from E5, E8, E15 and E18 retina showed a single band at 68kDa. Lower insert shows β-tubulin loading control. Specificity of pTAK1 antibody was tested by preabsorbing pTAK1 antibody with immunizing peptide and incubating E8 samples with untreated antibody (+) or preabsorbed antibody (pep) and detecting bands. Loading control for the experiment was β-tubulin. Scale bars in A = 50μm for images A-F.

**Supplemental Figure 3: Immunolabel with pTAK1 preabsorbed with immunizing peptide.**

Retinal sections from E3, E5, E8, E12, E15, and E18 chick embryos were co-labeled pTAK1 (A, D, G, J, M, P) and Hoechst nuclear stain (B, E, H, K, N, O). Overlap in labels is shown in C, F, I, L, O, R. Abbreviations: RPE, retinal pigmented epithelium; NBL, neuroblast layer; INL, inner nuclear layer; GCL, ganglion cell layer. Scale bar in A=50μm for images A-R

**Supplemental Figure 4: *Ex ovo* retinal cultures treated with TAK1 inhibitor Takinib showed a similar increase in proliferation and differentiation in comparison to 5Z-7-oxozeaenol.** E6 *ex-ovo* retinal cultures were treated with vehicle or 100pM Takinib for 24 hours and sections through fixed frozen retinas were quantitated for cleaved caspase 3 (CC3; A), phosphorylated JNK (pJNK; B), SOX2 (C), islet (D), or Visinin (E). Graphs are representations of the number of positive cells as a percentage of Hoechst-labeled cells (labels the nuclei of all cells) in each section. Statistical significance was determined using two-tailed t-tests (GraphPad Prism).

**Supplemental Figure 5: Measurements of retinal thickness in vehicle and TAKi-treated *ex ovo* cultures.** *Ex-ovo* retinas treated with vehicle or TAK1 inhibitor, Takinib, for 24 hours were fixed, cryosectioned, and thickness of the sectioned measured using Image J/Fiji. Thickness was measured from the outer edge of the retina where dividing progenitors are located to the nerve fiber layer at the inner edge of the retina. The average thickness of the vehicle- and TAKi-treated retinas is shown in A. The graph is a representation of the number of positive cells as a percentage of Hoechst-labeled cells (labels the nuclei of all cells) in each section. Statistical significance was determined using two-tailed t-tests (Graphpad Prism). Numbers of retinal sections that fell into 50µm increments of retinal thickness of vehicle- and TAKi-treated cultures are shown in B. Measurements from treated retinas were ranked from highest to lowest thickness and graphed as shown in C.
